# The Effect of Stimuli Level on Distortion Product Otoacoustic Emission in Normal Hearing Adults

**DOI:** 10.3390/acoustics5010005

**Published:** 2023-01-10

**Authors:** Maryam Naghibolhosseini

**Affiliations:** 1 Department of Communicative Sciences and Disorders, Michigan State University, East Lansing, MI 48823, USA

**Keywords:** DPOAE, Generator Component, Reflection Component, Input/Output Function, Cochlea Compression

## Abstract

The goal of this study is to compare three of the most commonly used primary-level relation paradigms (i.e., Scissors, Boys Town ‘Optimal’, and Equal-Level) in generation of distortion product otoacoustic emissions (DPOAEs) in normal hearing adults. The generator and reflection components were extracted from DPOAEs in each paradigm. The generator and reflection component levels and input/output (I/O) functions were compared across paradigms and primary-tone levels. The results showed a different I/O function growth behavior across frequency and levels among paradigms. The Optimal paradigm showed a systematic change in the generator and reflection component levels and I/O slopes across primary levels among subjects. Moreover, the levels and slopes in the Optimal paradigm were more distinct across levels with less variations across frequency leading to a systematic change in the DPOAE fine structure across levels. The I/O functions were found to be more sensitive to the selected paradigm; especially the I/O function for the reflection component. The I/O functions of the reflection components showed large variability across frequencies due to different frequency shifts in their microstructure depending on the paradigm. The findings of this study suggested the Optimal paradigm as the proper primary-level relation to study cochlear amplification/compression. The findings of this study shows that care needs to be taken in comparing the findings of different studies that generated DPOAEs with a different level-relation paradigm.

## 1. Introduction

Sound signals can be generated inside the cochlea by presenting two primary tones in the ear canal. The generated sounds travel back toward the ear canal, where they can be recorded by a sensitive microphone. The recorded emissions are called distortion product otoacoustic emissions (DPOAEs). DPOAEs are used to study the health and function of the cochlea [1–3] and the transmission characteristics of the outer and middle ear [4–7], also to differentiate between the middle and inner ear dysfunctions [8].

When measured with a high-frequency resolution, DPOAEs exhibit a quasi-periodic pattern of peaks and valleys across frequency, called the fine structure [9–12]. While DPOAEs are widely employed in clinics for hearing evaluation, their clinical use is mainly limited to the measurement of their level and signal to noise ratio (SNR) at only a few discrete frequencies [13]. Depending on where in the fine structure the frequencies are located, a maximum or minimum DPOAE level could occur, which makes DPOAE interpretation for hearing loss detection/diagnosis more challenging. Although the effectiveness of high-frequency resolution DPOAE has long been recognized in research, it has not been yet utilized in clinics. Therefore, there is a need for a standard protocol for obtaining high-frequency resolution DPOAE that will lead to the most clinically meaningful information. This makes the right selection of primary-tone levels and frequencies in DPOAE production critical.

It has been shown that the formation of fine structure patterns is due to the interaction of two main DPOAE components inside the cochlea, i.e., the generator and reflection components [11,14]. The two primaries *f*_1_/*L*_1_ (with frequency *f*_1_ and level *L*_1_) and *f*_2_/*L*_2_ (with frequency *f*_2_ and level *L*_2_), presented in the ear canal, travel toward the cochlea and they excite the basilar membrane (BM) at their best frequency places, where they overlap and due to their nonlinear interaction, mechanical waves are generated at different frequencies such as 2*f*_1_-*f*_2_. Each generated wave travels basally and can be recorded as the generator component in the ear canal. A part of the wave travels apically toward its own best place at 2*f*_1_-*f*_2_ in the cochlea while being amplified due to the cochlear nonlinear amplification, gets reflected back from its best place, and can be recorded as the reflection component in the ear canal. The DPOAE obtained in the ear canal is the vector summation of the generator and reflection components’ real and imaginary parts [11,14–16].

The constructive and destructive interactions of the two components lead to a formation of patterns of minima and maxima in DPOAEs. The locations of these extrema depend on the amplitudes and phases of the two components. The amplitudes of the two components depend on the extent of overlap of the two primaries inside the cochlea and the size of reflection from the distortion product place. The size of overlap region depends on the primary-level relation and frequency separation. Hence, the characteristics of primary tones play an important role in the level and phase of DPOAE [17,18].

Researchers have tested different primary-level relations and frequency ratios to determine the optimal levels/frequencies for obtaining DPOAEs [18–25]. Most studies selected a combination of *L*_1_/*L*_2_ that maximized the level of DPOAEs due to the importance of DPOAE level in determining the health of cochlea [18,23,24]. Several studies suggested an unequal-level paradigm with a constant level relation of *L*_1_ = *L*_2_+10 (15) *dB SPL* [19, 26]. Others proposed using level relations of *L*_1_ = 0.4*L*_2_+39 *dB SPL*, called the Scissors paradigm [27,28] and *L*_1_ = 0.45*L*_2_+44 *dB SPL* [23] based on the finding that the optimal difference between the primary-tone levels decreases as *L*_2_ increases [18,23,24,29]. These researchers selected the optimal level/frequency relations based on the DPOAE levels at discrete frequencies. Moreover, most studies did not look at the DPOAE components [18,20,23,27,30]. Zelle et al. searched for the Optimal paradigm at discrete frequencies while investigating the impact of the nonlinear distortion component on DPOAEs [24].

Other than DPOAE levels that provide critical information about the health of cochlea, the DPOAE input/output (I/O) function can be used as a biomarker to detect cochlear dysfunction [31]. It has been shown experimentally that the BM response grows nonlinearly at the best frequency place of the stimulus [32,33]. A similar nonlinear growth behavior has been observed in DPOAE I/O functions [34]. Accordingly, DPOAEs were utilized to study cochlear compressive nonlinear behavior in normal hearing and hearing impaired individuals [35–37], to compare with loudness growth curves [38], and to estimate auditory thresholds [27,39]. Hence, the DPOAE I/O function could be beneficial clinically, however, it has mostly been employed in research and not in clinics.

The generator and reflection component levels and I/O functions are all affected by the selection of primary-level relations and accordingly impact the DPOAE levels and I/O functions. Long et al. found frequency shifts in the points of maxima across levels in DPOAE fine structure using several level-relation paradigms [40]. They also compared the average generator and reflection component I/O functions across paradigms. Although differences between the average I/O functions were observed [40], it has not been investigated how the two components’ levels and I/O functions change depending on the frequency, levels, and primary-level relations. Understanding these effects is crucial in deciding what paradigm to use, how to compare data obtained using different paradigms, and better understanding the mechanisms of DPOAE generation and their effective utilization in studying cochlear health and function. The goal of this study is to compare the generator and reflection component levels and I/O growth behavior across three widely-used level-relation paradigms: Equal-Level (EL), Optimal (Op), developed by Neely et al., 2005, and Scissors (Sc). Moreover, the impact of generator and reflection components on DPOAE fine structure pattern across paradigms will be discussed. Although both the frequency and level relations play critical roles in determining the DPOAE signal characteristics, this study focuses on several level-relation paradigms while obtaining the DPOAEs with a high-frequency resolution.

## 2. Materials and Methods

### 2.1. Data Collection

DPOAE data were collected from five normal hearing adults (4 female, 1 male) with audiometric thresholds of better than 20 *dB* at octave frequencies between 0.25 – 8 *kHz* and normal tympanogram results. Logarithmically sweeping tones up in frequency (*f*_1_/*L*_1_ and *f*_2_/*L*_2_) with a duration of 6 seconds over 3 octaves (2s/octave) were used as primary tones. The primary tones were presented using the following primary-level relation paradigms: i) the Op paradigm, *L*_1_ = 0.45*L*_2_ + 44 *dB SPL*; ii) Sc paradigm, *L*_1_ = 0.4*L*_2_ + 39 *dB SPL* (*L*_1_ = *L*_2_ for *L*_2_ ≥ 65 *dB SPL*); and iii) EL paradigm, *L*_1_ = *L*_2_. The levels and frequencies of the primaries in different paradigms are shown in Table 1.

The DPOAE data were collected while subjects were seating on a reclining chair in a double-walled sound treated booth. ER2 insert earphones (Etymotic Research Inc., Elk Grove Village, IL, USA), connected to an ER10 probe microphone system (Etymotic Research Inc., Elk Grove Village, IL, USA), were used for the data collection. The captured signals were passed through an Etymotic preamplifier and a battery-operated Stanford Research Systems SRS 560 low-noise preamplifier (Stanford Research Systems, Sunnyvale, CA, USA). The output of SR560 was connected to MOTU 828 Firewire Audio Interface (Motu Inc., Cambridge MA, USA), which digitized the signal at a sampling rate of 44.1 kHz before it was stored on a Mac computer for offline analysis. The data analysis was conducted using MATLAB R2020b (MathWorks Inc., Natick, MA).

### 2.2. Data Analysis

The spectrograms of the ear canal recordings (aka sweeps) at each stimulus level were examined individually and those with high noise levels were manually removed. The remaining sweeps were averaged at each stimulus level to increase the signal to noise ratio. The recorded sweeps at each level were subtracted from each other and averaged to provide an estimate of the noise floor. A least squares fit analysis (using an overlapping Hann window) was applied to the averaged sweeps to estimate the phases and amplitudes of generator and reflection components, and the combined DPOAE [41,42]. The overlapping Hann window was moved along the data in the time-frequency domain. Although other DPOAE analysis methods in the time-frequency domain have been proposed (e.g., based on the wavelet transforms [43]), the least squares fit method is computationally more efficient. The least squares fit analysis with overlapping Hann windows was used in the current study due to its efficiency and robustness in analyzing the swept-frequency responses. This method analyzed the data based on the expected time-frequency parameters of the DPOAE/components with unknown magnitudes and phases within each window. This procedure worked by minimizing the least squared error between the recorded data (with known magnitudes and phases) and the estimated magnitudes and phases of the DPOAE/components. This method only evaluates the frequencies of interests and does not require the frequencies to fall at the center of the analysis window as for example the fast Fourier transform method does. Moreover, the spectral leakage using this method is minimal. The least squares fit method also allows for the dynamic fitting of the delay of a given component leading to more accurate estimation of the components [41,42]. The center frequency of the band pass filters changed as the DPOAE frequency changed. A wide-band filter with 5512 samples (i.e., 8 *Hz* bandwidth) with a step size of 551 was applied to extract the combined DPOAE. A narrow-band filter with 22050 samples (i.e., 2 *Hz* bandwidth) with a step size of 2205 was utilized next to separate the generator and reflection components. A frequency-dependent latency function was used to separate the two components due to the delay in production of the reflection component with respect to the generator component [41,44]. The latency of the generator component was fixed and the latency of the reflection component was an empirically-derived latency function, which was an inverse of a 2^*nd*^ order polynomial [42,45].

The levels of the generator and reflection components across frequency at different *L*_2_s were compared between paradigms. The I/O functions were compared at low, mid, and high *L*_2_s. To do so, a piecewise linear function with two breakpoints (at *L*_2_ = 40 and 65 *dB SPL*) was fitted on the generator and reflection component I/O functions at each frequency. Subsequently, the generator and reflection component level growth at low *L*_2_s (i.e., *L*_2_ = 25 – 40 *dB SPL*), mid levels (i.e., *L*_2_ = 40 – 65 *dB SPL*), and high levels (i.e., *L*_2_ = 65 – 75 *dB SPL*) were compared across subjects and paradigms. Analysis of Variance (ANOVA) was used to evaluate the differences among the paradigms. For the comparison of the paradigms, the null hypothesis stated there was no significant difference in the means of levels/slopes of the components at different *L*_2_s between the paradigms. The comparison of the levels/slopes at different *L*_2_s within each paradigm was done with the null hypothesis being that no difference was observed at different *L*_2_s within each paradigm. Since there were more than two groups (paradigms), we corrected for multiple comparisons using the Tukey post-hoc test. We investigated the distributions of the outcomes; no extreme outliers were detected, and the ANOVA assumptions seem to hold. In addition, we confirmed our results whenever necessary using the positional mean (Median) and the non-parametric test of Kruskal-Wallis. Lastly, the impact of the generator and reflection component levels on the observed DPOAE fine structure in different paradigms was discussed.

## 3. Results

### 3.1. Generator Component

**Figure 1.**
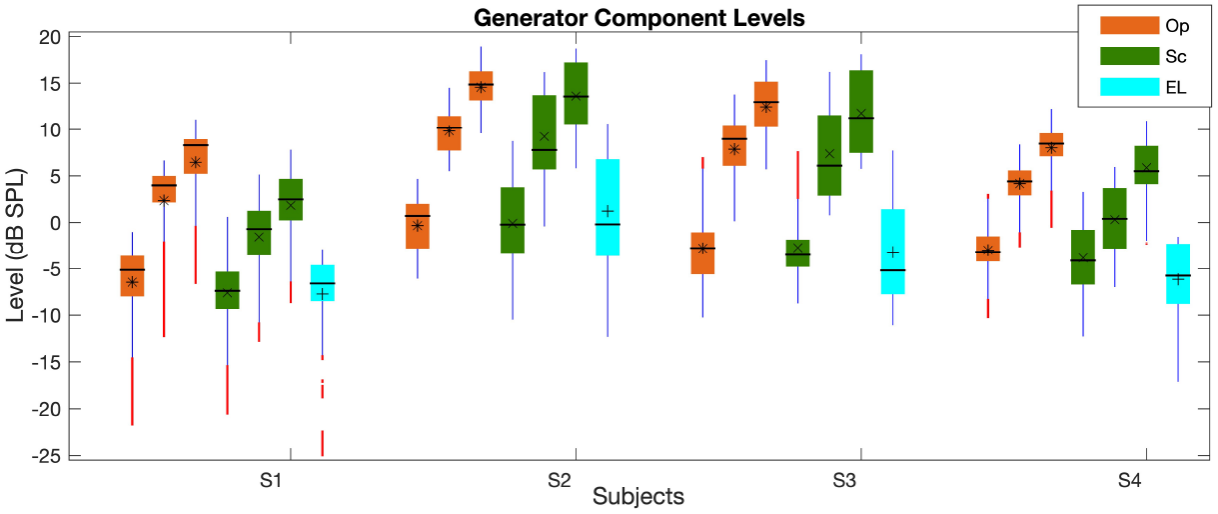
Boxplot of the *L*_*gen*_ averaged at low, mid, and high *L*_2_*s* (three orange and green bars in the Op and Sc paradigms, respectively; the cyan bars show the mid-level values in the EL paradigm) across frequency. The central horizontal line, and the bottom and top edges of the boxes indicate the median, 25^*th*^ and 75^*th*^ percentiles, respectively. The averages of individual bars are indicated by the *, ×, + symbols (for the Op, Sc, and EL paradigms, respectively) inside the boxes. The whiskers extend to the most extreme data points and outliers are plotted using red dots. The subjects’ IDs (S1-S4) are indicated on the x-axis.

The levels of the generator component (*L*_*gen*_) at low, mid and high *L*_2_s at frequencies with SNR of higher than 6 *dB SPL* are shown in Figure 1. The different paradigms are shown by different colors as indicated in the legend. At each frequency, the *L*_*gen*_ was averaged over *L*_2_ = 25 − 45 *dB SPL* (low levels), *L*_2_ = 45 − 65 *dB SPL* (mid levels) and *L*_2_ = 65 − 75 *dB SPL* (high levels); the three bars for each subject (S1-S4) from left to right belong to low, mid, and high levels, respectively. The cyan bar shows the *L*_*gen*_ for the EL paradigm at mid levels; note that the *L*_*gen*_s at high levels of the EL paradigm is the same as those of the Sc paradigm and hence, were not plotted. As can be seen in Figure 1, the *L*_*gen*_ increases from low to high *L*_2_s in all paradigms. The Sc paradigm shows higher variability across frequency in comparison with the Op paradigm at mid and high *L*_2_ levels. There is also less overlap between *L*_*gen*_s at different levels across frequency in the Op paradigm. Moreover, the average *L*_*gen*_s are higher in the Op paradigm than in the Sc for all *L*_2_ ranges. The *L*_*gen*_s for the EL paradigm at mid levels are in the same range as those of the Op and Sc paradigms at low levels but show more variability across frequency.

**Figure 2.**
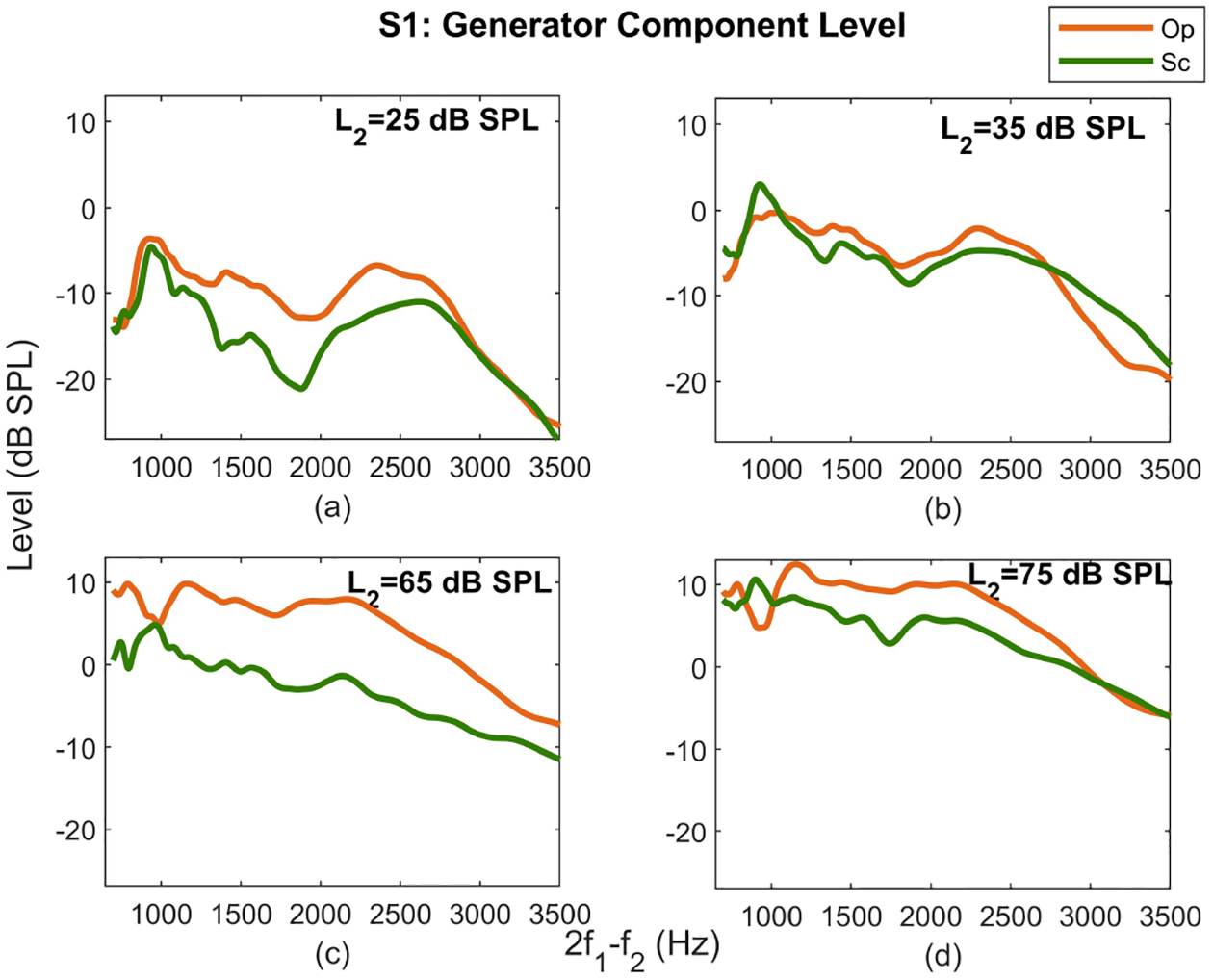
*L*_*gen*_ as a function of frequency at *L*_2_ = 25 *dB SPL* (panel (a)), 35 *dB SPL* (panel (b)), 65 *dB SPL* (panel (c)), and 75 *dB SPL* (panel (d)) for the Op (orange curves) and Sc (green curves) paradigms for subject S1.

In addition to the comparison of *L*_*gen*_s at different *L*_2_ ranges, the *L*_*gen*_s as a function of frequency were also compared at individual levels across paradigms. Figure 2 shows *L*_*gen*_s at several *L*_2_s (indicated inside each graph) in the Op (orange curves) and Sc (green curves) paradigms. As can be seen, *L*_*gen*_ in the Op paradigm is always higher than the Sc at mid frequencies for different *L*_2_s. A similar observation for the Op and Sc paradigms was made for the other subjects at all *L*_2_s.

**Figure 3.**
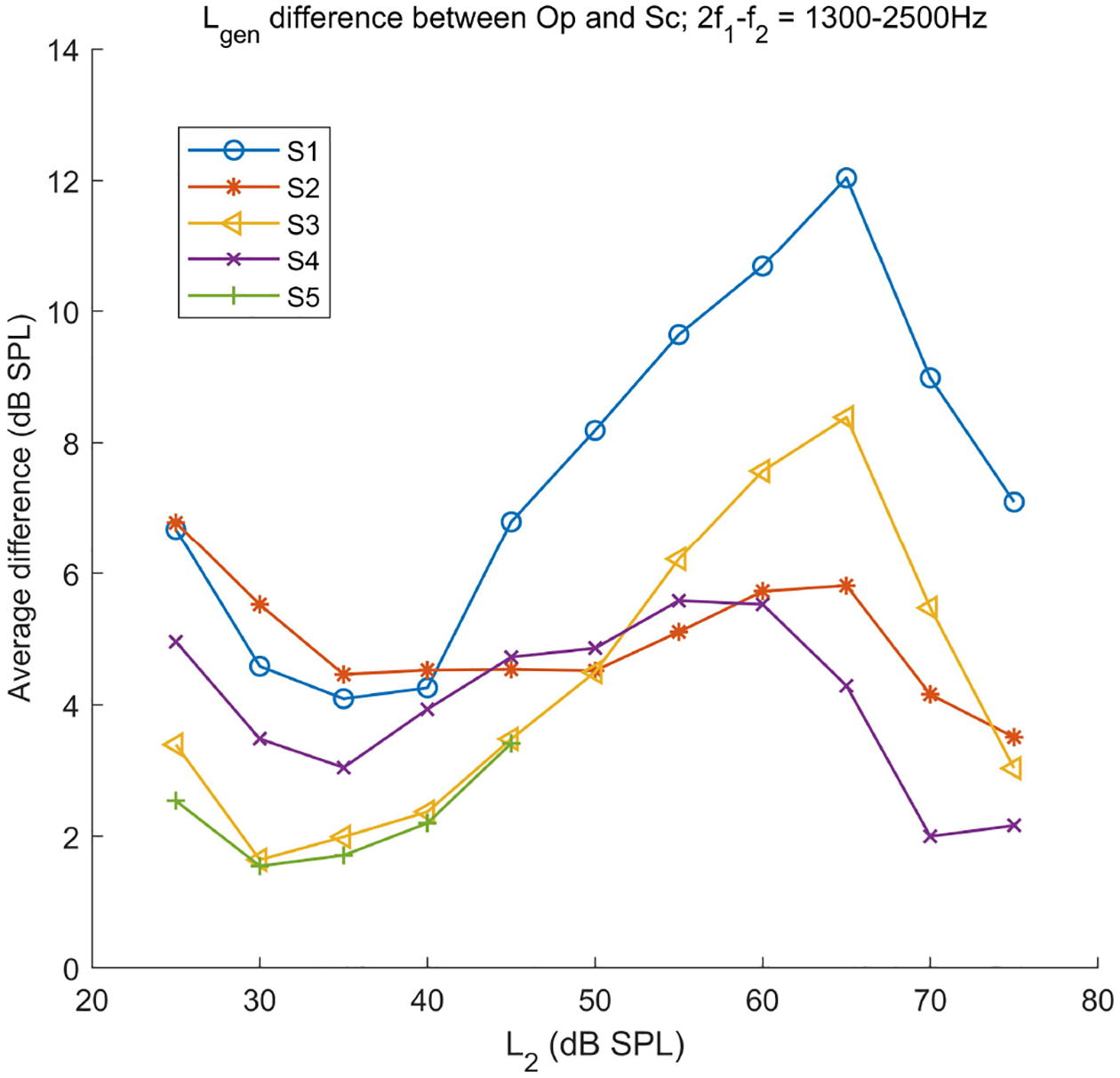
Average *L*_*gen*_ difference between the Op and Sc paradigms across *L*_2_ at the frequency range of 1300 − 2500 *Hz* for different subjects (shown in the legend).

The average difference of *L*_*gen*_s between the Op and Sc paradigms at individual *L*_2_s at mid frequencies (1300 − 2500 *Hz*) are shown in Figure 3. Each curve shows the difference for a different subject as indicated in the legend. As can be seen in this figure, the *L*_*gen*_ difference at mid-frequencies decreases as *L*_2_ increases from 25 to 30 – 35 *dB SPL*, then increases as *L*_2_ goes up to 60 − 65 *dB SPL*, and decreases again as *L*_2_ increases toward 75 *dB SPL* systematically.

The relationship between the *L*_*gen*_ of the Op and Sc paradigms was different at low and high frequencies. At low frequencies, the *L*_*gen*_ of the Op paradigm was higher than that of the Sc paradigm at *L*_2_ = 60 − 65 *dB SPL* for all subjects. However, at other *L*_2_s, the *L*_*gen*_ of the Sc paradigm was either higher or lower than that of the Op at different low and high frequencies. The comparison of the *L*_*gen*_ between the EL and Sc/Op paradigm showed that as *L*_2_ increased, the average difference between *L*_*gen*_s of the EL and Op/Sc paradigms decreased almost linearly in contrast to what is observed for the difference between the Op and Sc in Figure 3.

Tukey’s test showed that the *L*_*gen*_s in the EL paradigm at mid levels were statistically significantly different (*p* < 0.05) from those in the Op and Sc paradigms at mid and high-levels but no statistically significantly difference was observed with the Op/Sc slopes at low levels. Also, statistically significant difference was observed between the EL paradigm at mid and high levels. In the Sc and Op paradigm, statistically significant difference between the low and high levels within each paradigm was observed for the *L*_*gen*_s.

**Figure 4.**
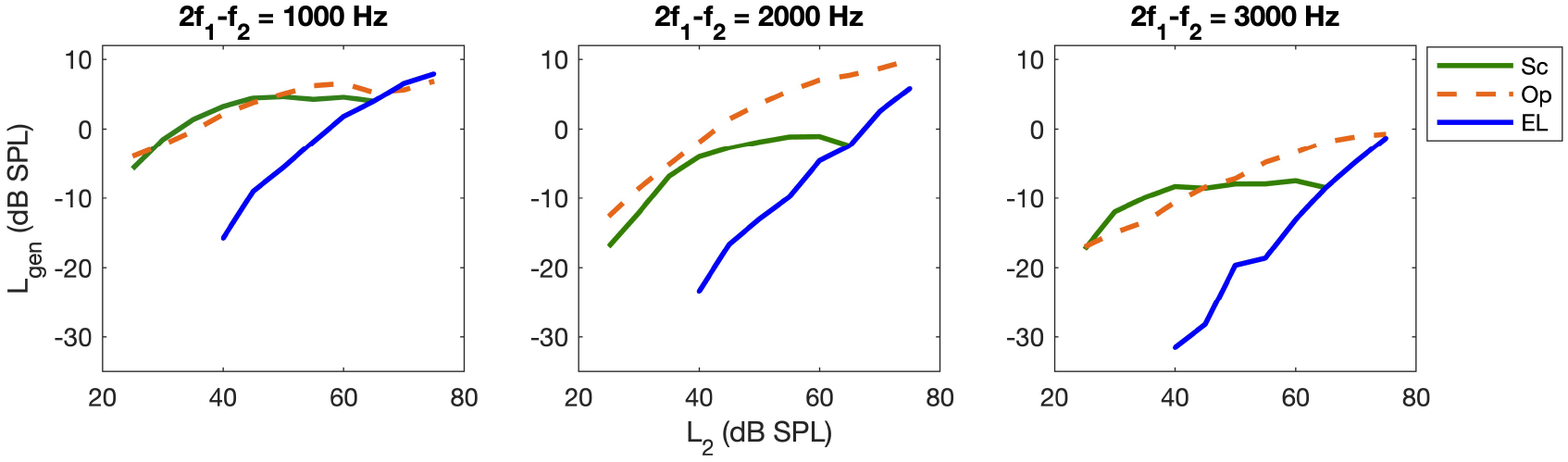
*I*/*O*_*gen*_ at frequencies of 1000, 2000, 3000 *Hz* for different paradigms (shown in the legend) for S1.

The generator component I/O function (*I*/*O*_*gen*_) at frequencies of 1000, 2000, and 3000 *Hz* for the different paradigms for subject S1 are shown in Figure 4. As can be seen, the slopes and the output levels of the I/O function changes for the different paradigms across frequency. Accordingly, to better study the variations of the I/O functions across frequency, in addition to looking at the output levels at low, mid, and high *L*_2_s, as explained above, the variations of the slopes at low, mid, and high *L*_2_s across frequency will be shown and discussed in this section.

**Figure 5.**
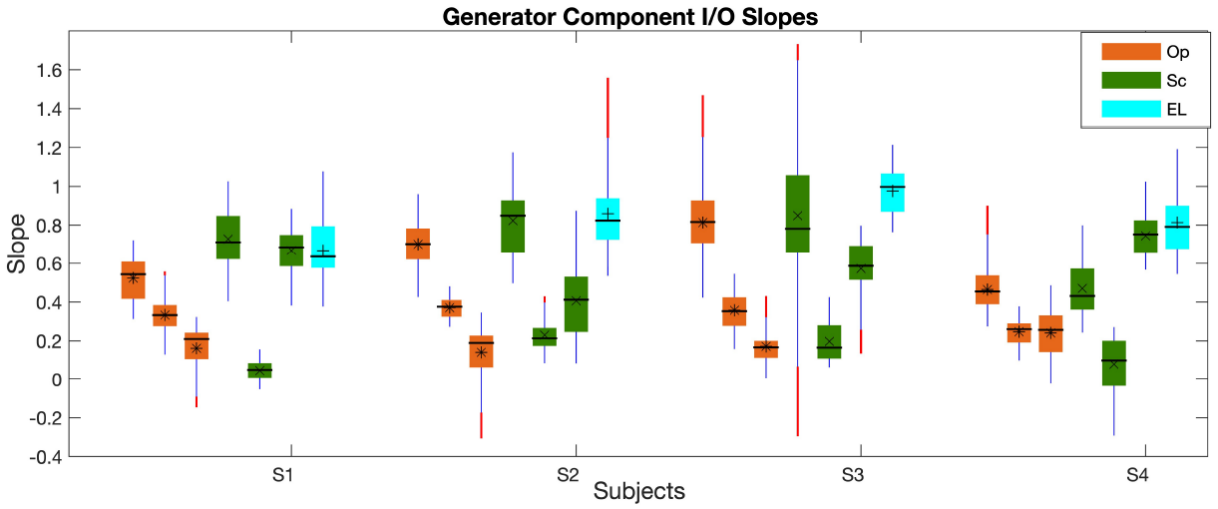
Boxplot of the *I*/*O*_*gen*_ slopes at low, mid, and high *L*_2_*s* (three orange and green bars in the Op and Sc paradigms, respectively; the cyan bars show the mid-level values in EL paradigm) across frequency. The central horizontal line, and the bottom and top edges of the boxes indicate the median, 25^*th*^ and 75^*th*^ percentiles, respectively. The averages of individual bars are indicated by the *, ×, + symbols (for the Op, Sc, and EL paradigms, respectively) inside the boxes. The whiskers extend to the most extreme data points and outliers are plotted using red dots. The subjects IDs (S1-S4) are indicated on the x-axis.

Figure 5 shows the slopes of the *I*/*O*_*gen*_ at low, mid, and high *L*_2_s, calculated at each frequency using the piecewise linear function, explained in the Methods section. As can be seen, the *I*/*O*_*gen*_ slopes are more distinct from each other than the *L*_*gen*_s across paradigms. The highest slopes at low and high levels belong to the Sc paradigm but this paradigm shows the shallowest slope at mid levels. The highest slopes at mid levels are seen in the EL paradigm and they are more comparable to the slopes of the Op/Sc paradigms at low levels. The *I*/*O*_*gen*_ slopes in the Op paradigm show a similar trend of a constant decrease as *L*_2_ increases across subjects. The Sc paradigm shows a large reduction in the slope from low to mid levels and a high increase in the slope from mid to high levels. In fact, in the Sc paradigm, the high-level *I*/*O*_*gen*_ slopes are closer to the low-level slopes than to the mid-level slopes; the high-level slopes are even higher than the low-level slopes for S4. Moreover, the *I*/*O*_*gen*_ slope is more dispersed across frequency in the Sc paradigm in comparison with the Op paradigm at respective levels. The EL paradigm shows the highest dispersion of slopes at mid levels.

**Figure 6.**
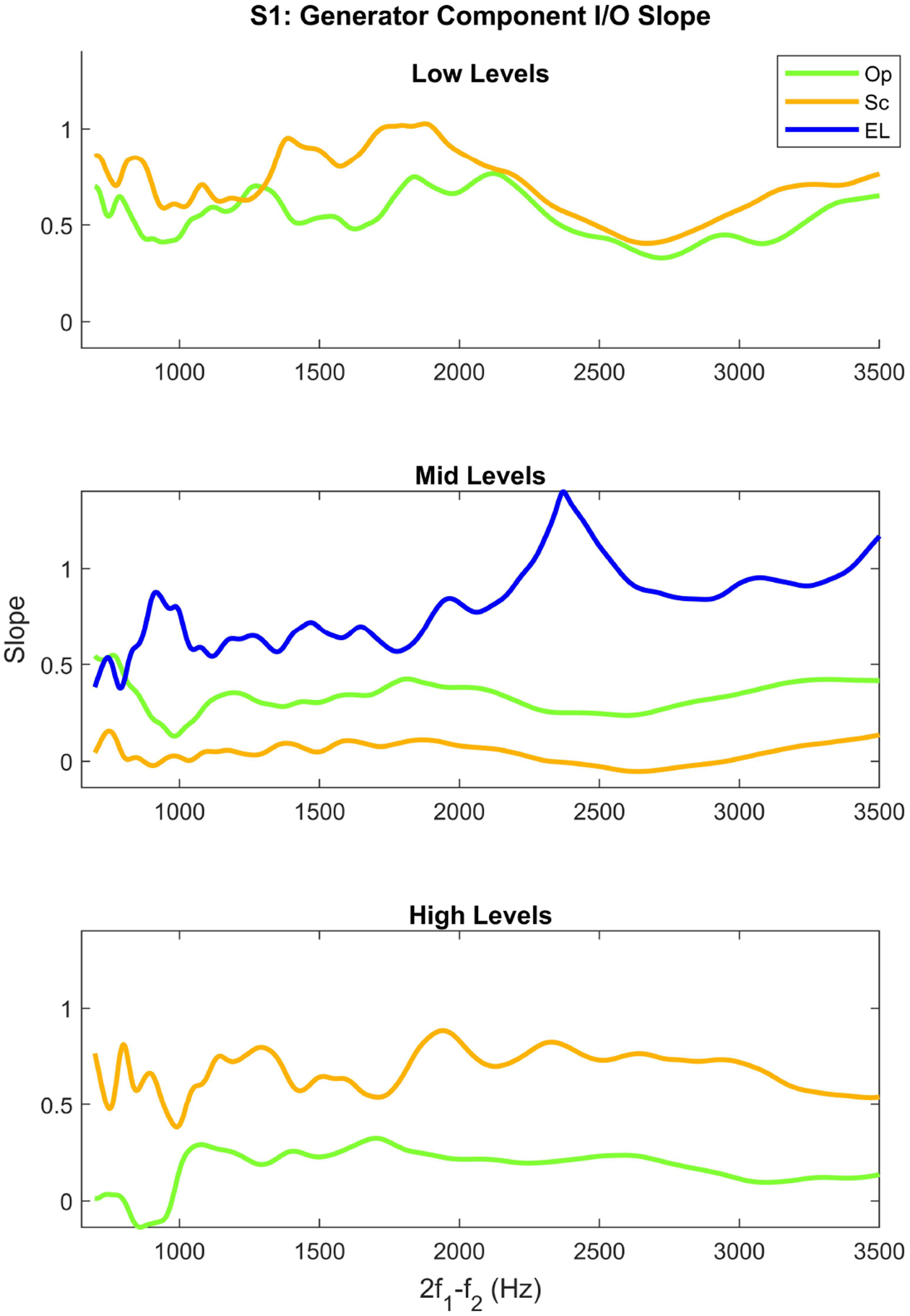
*I*/*O*_*gen*_ slope at low, mid, and high *L*_2_*s* in the Op (orange), Sc (green), and EL (blue) paradigms across frequency for subject S1.

Figure 6 shows the *I*/*O*_*gen*_ slope across frequency at low (panel (a)), mid (panel (b)), and high levels (panel (c)) for one subject. As can be seen, the Sc paradigm shows a higher slope than the Op across frequency at low and high levels. However, the slope of the Op paradigm is higher than that of the Sc at mid levels. The EL paradigm shows the highest slope with the largest variability across frequency at mid levels. The large peak around 2500 *Hz* in the EL paradigm at mid levels is due to the large noise level around this frequency. The *I*/*O*_*gen*_ slope in the Sc paradigm shows the highest variability across frequency at all levels. The *I*/*O*_*gen*_ slopes for all paradigms become more smooth toward higher frequencies. Similar observations were made for other subjects.

The correlation of the *I*/*O*_*gen*_ slopes at low, mid, and high levels across frequency were variable between paradigms and subjects. The main observation was that the correlation between the slopes reduced as *L*_2_ increased. The correlation between *L*_*gen*_s at low, mid, and high levels was always above 0.5; but much lower correlations (and even negative values) between the slopes were observed.

Tukey’s test showed that the *I*/*O*_*gen*_ slopes in the EL paradigm at mid levels were statistically significantly different (*p* < 0.05) from those in the Op and Sc paradigms at mid and high-levels but no statistically significantly difference was observed with the Op/Sc slopes at low levels. The slopes in the Sc paradigm were statistically significantly different at low, mid, and high *L*_2_s. The Op paradigm showed statistically significant difference between the slopes at low and high levels. Moreover, the slopes in the Op and Sc paradigms at high levels were statistically significantly different. Also, statistically significant difference was observed between the EL paradigm at mid and high levels.

### 3.2. Reflection Component

**Figure 7.**
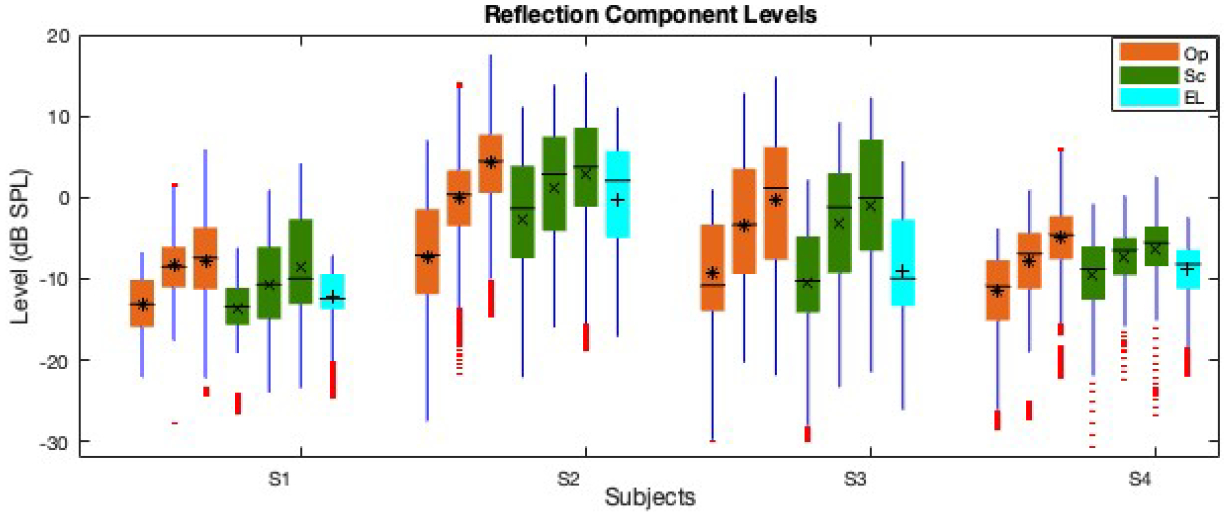
Boxplot of the *L*_*re f l*_ averaged at low, mid, and high *L*_2_*s* (three orange and green bars in the Op and Sc paradigms, respectively; the cyan bars show the mid-level values in the EL paradigm) across frequency. The central horizontal line, and the bottom and top edges of the boxes indicate the median, 25^*th*^ and 75^*th*^ percentiles, respectively. The averages of individual bars are indicated by the *, ×, + symbols (for the Op, Sc, and EL paradigms, respectively) inside the boxes. The whiskers extend to the most extreme data points and outliers are plotted using red dots. The subjects IDs (S1-S4) are indicated on the x-axis.

The boxplot of the reflection component level (*L*_*re f l*_) averaged at low, mid, and high *L*_2_s at frequencies with SNR of higher than 6 *dB SPL* is shown in Figure 7. As can be seen in the figure, the *L*_*re f l*_ level increases from low to high levels in all paradigms. The *L*_*re f l*_ values across frequency overlap for the different paradigms. The EL paradigm shows slightly lower *L*_*re f l*_s at mid levels than those of the Op/Sc. Moreover, the *L*_*re f l*_ dispersion across frequency for different paradigms is much larger than that of the *L*_*gen*_ (Figure 1).

**Figure 8.**
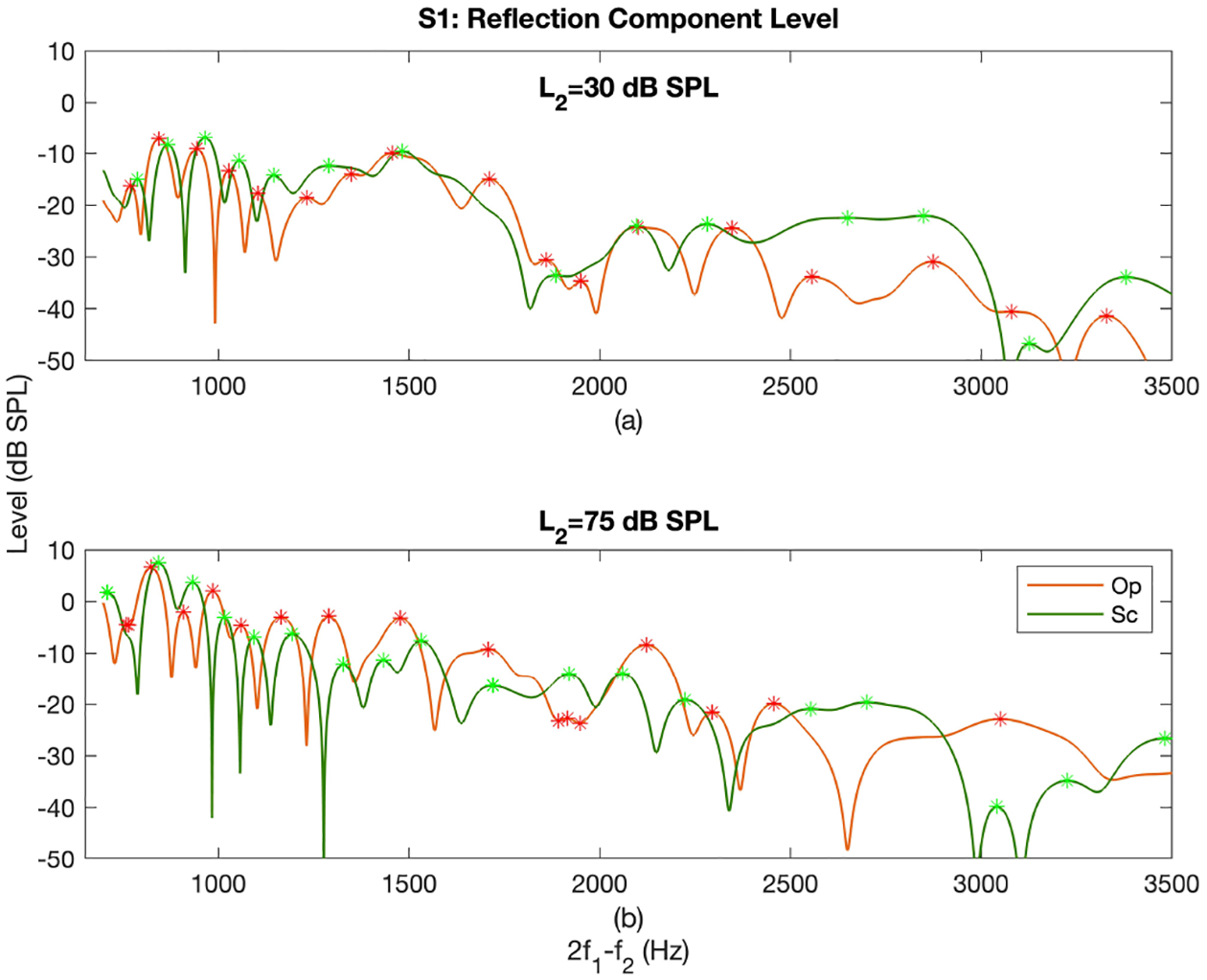
*L*_*re f l*_ as a function of frequency at *L*_2_ = 30 *dB SPL* (panel (a)) and *L*_2_ = 75 *dB SPL* (panel (b)) in the Op (orange curves) and Sc (green curves) paradigms for subject S1. The red and green stars indicate the maxima in the Op and Sc curves, respectively.

The *L*_*re f l*_ as a function of frequency at *L*_2_ = 30 *dB SPL* (panel (a)) and 75 *dB SPL* (panel (b)) in the Op (orange curves) and Sc (green curves) paradigms is shown in Figure 8. The maxima are indicated with the stars on both curves. As can be seen, the *L*_*re f l*_ patterns are very similar across frequency but there is a lag in the location of maxima and minima across paradigms. The amount of frequency lag changed as a function of frequency and *L*_2_. Moreover, the frequencies of maxima were the lowest in the Op paradigm and were the highest in the EL paradigm across participants. Due to the lower SNR at higher frequencies, the two curves become less aligned at higher frequencies. The frequencies of maxima decreased as *L*_2_ increased in all paradigms (see Figure 8 as an example for the Op and Sc paradigms at two levels). Additionally, high correlations between the *L*_*re f l*_ of different paradigms were observed at all individual *L*_2_s. The *L*_*re f l*_ at individual *L*_2_s were compared across paradigms visually considering the minima/maxima points were aligned. Accordingly, the *L*_*re f l*_ was often the lowest for the EL paradigm across participants. The *L*_*re f l*_s for the Op and Sc paradigm were lower or higher than each other at different frequencies and *L*_2_. The difference between the levels across paradigms reduced as *L*_2_ increased.

The *L*_*re f l*_s showed higher correlations than the *L*_*gen*_ between the Op and EL paradigms. A high correlation was also observed between the Op and Sc paradigms except for S1, which interestingly also showed the highest *L*_*gen*_ difference between the Op and Sc paradigms at mid frequencies (see Figure 3). However, the *L*_*re f l*_ showed lower correlations than the *L*_*gen*_s between the Sc and EL paradigms.

**Figure 9.**
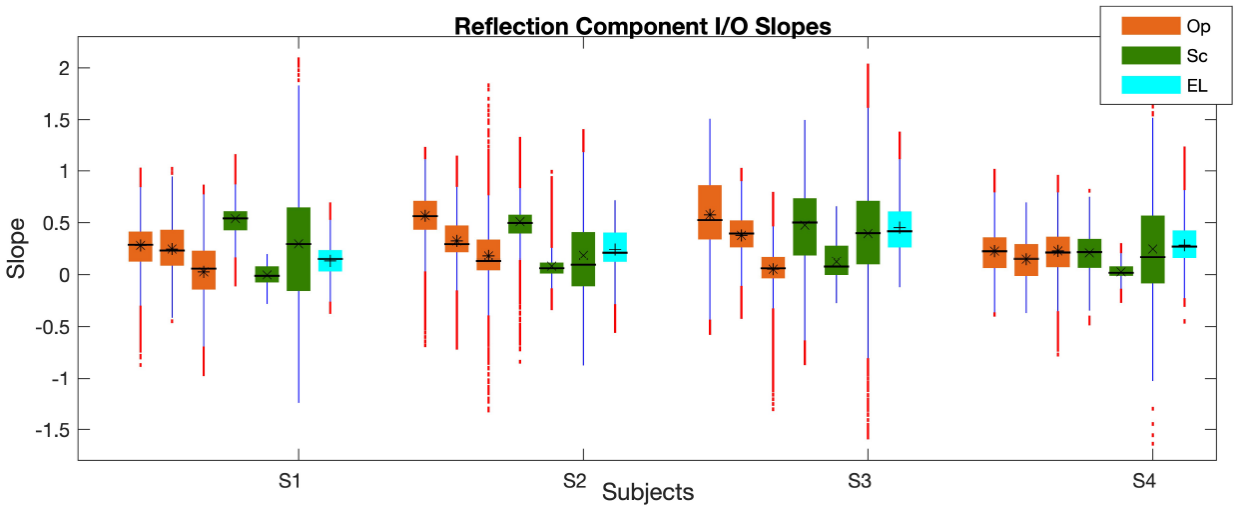
Boxplot of the *I*/*O*_*re f l*_ slopes at low, mid, and high *L*_2_*s* (three orange and green bars in the Op and Sc paradigms, respectively; the cyan bars show the mid-level values in the EL paradigm) across frequency. The central horizontal line, and the bottom and top edges of the boxes indicate the median, 25^*th*^ and 75^*th*^ percentiles, respectively. The averages of individual bars are indicated by the *, ×, + symbols (for the Op, Sc, and EL paradigms, respectively) inside the boxes. The whiskers extend to the most extreme data points and outliers are plotted using red dots. The subjects IDs (S1-S4) are indicated on the x-axis.

The boxplot of the reflection component I/O function (*I*/*O*_*re f l*_) slopes at low, mid, and high *L*_2_s, calculated at each frequency using the piecewise linear function is shown in Figure 9. As can be seen, the differences between the I/O slopes are more prominent than between the *L*_*re f l*_ in Figure 7; also, the large number of outliers across frequency is noticeable in Figure 9. The variability of the *I*/*O*_*re f l*_ slopes across frequency is the smallest for the Sc paradigm at mid levels and is the highest for the Sc paradigm at high levels. The I/O slope reduces as the level increases in the Op paradigm and shows a plateau in *L*_*re f l*_ at high levels as the slope becomes closer to zero except for S4. Subject S4 shows a much a similar slope across different *L*_2_ ranges. The slopes show clear reductions from low to mid levels in the Sc paradigm and come close to zero (saturate) at mid levels. The slopes at high levels are widely dispersed in the Sc paradigm and overlap with the slopes at low and mid levels at many frequencies. As can be seen in the EL paradigm, the slopes have overlap across many frequencies at mid and high levels. At mid levels, the slope is higher in the Op paradigm in comparison with the Sc. The correlations of the I/O slopes between the different paradigms were lower than 0.4 with some negative values.

## 4. Discussion

This study compared three of the most widely used primary-level relation paradigms (i.e., Equal-Level, Optimal, and Scissors) in production of DPOAEs. The generator and reflection component levels and I/O functions growth behavior were compared across paradigms. Due to the cochlear nonlinearity and BM skewed response to sound waves, the size of the overlap region of the primaries inside the cochlea is very sensitive to the level relations of the primaries. This mainly affects the nonlinear distortion component that is generated in the overlap region and subsequently the reflection component as it was observed in this study.

Although the average *L*_*gen*_s in the Op paradigm were higher than the Sc paradigm, comparing the *L*_*gen*_s at individual *L*_2_s showed higher *L*_*gen*_s in the Op paradigm only at mid frequencies. This showed that the higher overlap region in the Op paradigm only occurred at mid frequencies. The *L*_*gen*_ difference between the Op and Sc paradigms at mid frequencies showed a similar behavior across *L*_2_ for different participants (see Figure 3). This similar behavior was backed up by the results of the comparisons of *I*/*O*_*gen*_ between the two paradigms. The Sc paradigm showed higher *I*/*O*_*gen*_ slopes at low and high levels and accordingly lead to a decrease in the difference between the *L*_*gen*_s in the Op and Sc paradigms at low and high *L*_2_s. Moreover, the *I*/*O*_*gen*_ in the Sc paradigm showed larger saturation at mid *L*_2_s than in the Op, which lead to the increase in the difference between the *L*_*gen*_s at mid levels.

When comparing *L*_*gen*_s between the Op and Sc paradigms at low and high frequencies, different behaviors from mid frequencies (explained above) were observed. The *L*_*gen*_ was either lower or higher in the Op paradigm at different low and high frequencies. The *L*_*gen*_ and *I*/*O*_*gen*_ growth behavior were more variable at low frequencies while they got smoother as the frequency increased. This could be explained based on the different behavior of the BM at low and high frequencies; it has been observed that the bandwidth (aka the sharpness of tuning) of the BM responses decrease as the stimuli frequencies increase [33,46]. Hence, the bandwidth of the BM response is widened at low frequencies and it encompasses more adjacent natural modes of vibrations, accordingly the superposition of these modes would naturally give rise to a more wiggly/less-wavy nonlinear distortion component. At high frequencies, the distances between the natural frequencies/modes get closer than at low frequencies such that less of the adjacent natural frequencies are superimposed to generate the cochlear response. Hence, the resulting nonlinear combination of those modes would give birth to a more organized and wavy generator component. It should also be noted that the noise floor was higher at low frequencies, which might have played a role. The *L*_*gen*_ SNR was lower at higher frequencies, however, the smoother *L*_*gen*_ and *I*/*O*_*gen*_ at higher frequencies indicate that the lower SNR may not have played a role.

The *L*_*gen*_ in the EL paradigm was way below those of the Op and Sc paradigms with a much steeper slope. Hence, the difference between the *L*_*gen*_ of the EL and Op/Sc decreased constantly as *L*_2_ increased. The much lower *L*_*gen*_s in the EL paradigm is in line with previous studies that the overlap region of the primaries in the cochlea is smaller when using equal primary-tone levels in comparison with unequal levels [19,26]. The *L*_*gen*_s and *I*/*O*_*gen*_ slopes in the EL paradigm at mid levels were in the range of the Op and Sc paradigms at low levels. Tukey’s test showed that statistically significant difference existed in the *L*_*gen*_s and *I*/*O*_*gen*_ slopes between the EL paradigm at mid levels and the mid-level Op/Sc values but not between the EL paradigm at mid levels and the low-level ones in the Op/Sc paradigms. Hence, if the generator components by the EL and Sc/Op paradigms are compared between different studies, the comparison of *L*_*gen*_s of the EL at mid levels with the low-level *L*_*gen*_s in the Op/Sc paradigm should be considered.

The *L*_*gen*_ in the Op paradigm showed more distinct values at low, mid, and high *L*_2_s with a systematic increase as *L*_2_ increased. Additionally, a systematic decrease in the slope of the *I*/*O*_*gen*_ from low to high levels in the Op paradigm was observed. The Op paradigm showed the lowest variability in *L*_*gen*_s and *I*/*O*_*gen*_ slopes. The *I*/*O*_*gen*_ slopes in the Sc paradigm were statistically significantly different at low, mid, and high levels. More different behavior in the *I*/*O*_*gen*_ slopes across levels was observed in the Sc paradigm among subjects than was observed for the Op paradigm. Although individual differences are expected between subjects but too much variability is not favorable within a normal hearing group of participants. Considering the highest variability of the EL paradigm in *L*_*gen*_ than the other paradigms, the use of this paradigm may not be ideal.

The much lower correlations between the *I*/*O*_*gen*_ slopes than the *L*_*gen*_s show the higher sensitivity of the slopes to the selected paradigm. This signifies the difference in the generator component growth behavior across frequency in different paradigms and how selecting the right paradigm can impact the findings of a study. Another benefit of the Op paradigm was its ability to better explain the cochlear compressive behavior. The reduction of the *I*/*O*_*gen*_ slopes in the Op paradigm with *L*_2_’s increase was more in line with the cochlear compressive behavior that less amplification is provided for higher level sounds [47]. In contrast to the Op paradigm, the Sc paradigm showed a large decrease in the *I*/*O*_*gen*_ slope from low to mid levels and then an increase from mid to high levels; the slopes at high levels were even comparable or larger than the low-level slopes for some subjects. Hence, using the Op paradigm to study cochlear function is more favorable. Moreover, the Op paradigm showed a similar decreasing behavior in the *I*/*O*_*gen*_ slopes as *L*_2_ increased across subjects but the Sc paradigm showed different behaviors across subjects as explained earlier. This showed the potential of the Op paradigm in having more similarities between normal hearing subjects; yet different values across subjects capturing individual differences in cochlear mechanics and characteristics.

As explained above, the generator component levels and growth behavior were affected by the primary-level relation paradigms due to the direct impact of the paradigm on the generation site (overlap region) of the distortion component. Although the reflection component depends on the amount of energy generated in the overlap region as the apical wave generated in the overlap region travels to the reflection site, the reflection component is mainly affected by random cochlear irregularities and micromechanical impedance perturbations at the reflection site [16]. Accordingly, it was expected to observe a more systematic change on the reflection component across paradigms and perhaps less effect of paradigm change. Higher correlations were observed in the *L*_*re f l*_ at individual *L*_2_s between the Op and Sc/EL paradigms than between the *L*_*gen*_s, which supported this speculation. The *L*_*re f l*_ values had much larger overlap across paradigms than the *L*_*gen*_s, which supported our earlier statement.

**Figure 10.**
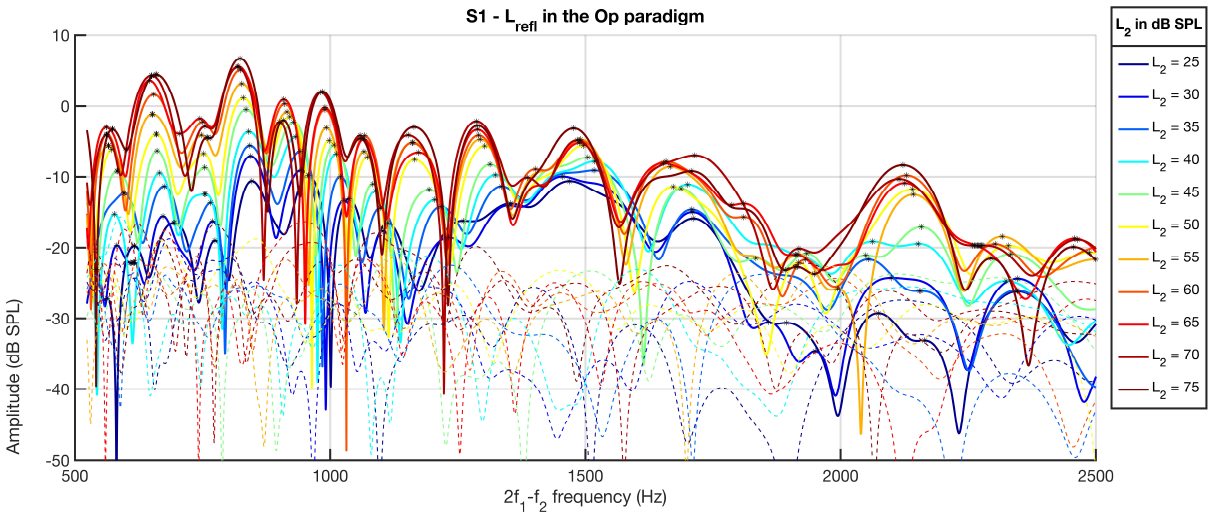
*L*_*re f l*_ across *L*_2_s shown in different colors; the stars indicate the location of maxima.

The main differences between the *L*_*re f l*_ of the different paradigms were in the location of maxima and minima. The minima/maxima frequencies in the Op paradigm preceded those of the Sc and EL paradigms; the minima/maxima in the EL paradigm occurred at higher frequencies. Figure 10 shows *L*_*re f l*_s for one subject in the Op paradigm across *L*_2_; the maxima are shown with black stars. The frequencies of maxima/minima decreased as *L*_2_ increased systematically in the Op paradigm (see Figure 10) and less systematically in the Sc and EL paradigms. This systematic decrease was not observable at higher frequencies, where the SNR reduced. The formation of the minima in the reflection component, leading to the microstructure pattern, is due to the out of phase interactions of the wavelets backscatterred from random spatial irregularities in the reflection site [16,48]. The more systematic changes in the *L*_*re f l*_ in the Op paradigm could be due to the more systematic patterns of *L*_*gen*_ across *L*_2_ in this paradigm. The apically traveling wave from the overlap region is impacted by cochlear nonlinear amplification en route before getting to the reflection site [9], hence, considering the more variability in the *L*_*gen*_ in the Sc and EL paradigms, the less systematic *L*_*re f l*_ pattern was expected. It should be noted that all participants except for S1 had spontaneous otoacoustic emissions (SOAEs) at one to five main frequencies. Although the impacts that SOAEs have on the reflection component may not be responsible for the observed differences among paradigms, but they lead to the observed differences in the reflection component among participants.

The *I*/*O*_*re f l*_ slopes at low, mid, and high levels showed large variability across frequency. The main reason was due to the different frequency shifts in the locations of maxima/minima across *L*_2_s between paradigms. Accordingly, the correlations between the *I*/*O*_*re f l*_ slopes of the different paradigms were very low and even negative as expected. The frequency shifts in maxima locations across *L*_2_ in the microstructure of the reflection components were larger in the Op paradigm than the Sc paradigm. This observation was in line with what was found by Long et al. about the frequency shifts in the DPOAE fine structure [40]. Long et al. found a downward frequency shift as *L*_2_ increased with the maximum shift (averaged across frequency) in the EL paradigm and the minimum shift in the Sc paradigm. This shows the impact of the reflection component amplitude microstructure on the DPOAE pattern and the importance of separating the two components for more accurate investigation of the cochlear function.

Large variability in the slope of the DPOAE I/O function across frequency has been observed and using the generator component I/O function is beneficial as it reduces this variability and leads to a more consistent I/O function across frequency [49]. Although the *I*/*O*_*re f l*_ growth can be beneficial as well, the frequency shifts in *L*_*re f l*_ across *L*_2_ should be considered in the interpretation of the results. Accordingly, in future work, it will be useful to use machine-learning based measures of similarity to compare the reflection components of the different paradigms. Researchers have studied DPOAE I/O function in normal hearing and hearing-impaired individuals at discrete frequencies [50–52]. The DPOAE I/O function at discrete frequencies depends on whether we are near a minimum or maximum. As was shown in the present study, even the generator and reflection component I/O functions vary across frequency and paradigms. Therefore, it is important to use high-frequency resolution DPOAEs while looking at DPOAE components to provide essential information about the cochlea amplification/compression characteristics.

Although it was reported previously that the magnitude of reflection component does not depend on the level-relation paradigm ([40]), this study still found some differences. Other than the frequency shift that was explained above, the *L*_*re f l*_ was mostly the lowest in the EL paradigm but for the Op and Sc paradigm, whether one was larger than the other depended on the frequency, levels and the subject. This observation could be related to the *L*_*gen*_ in different paradigms. The difference between the *L*_*re f l*_ of different paradigms reduced as *L*_2_ increased and as *L*_*re f l*_ got saturated. Additionally, the *I*/*O*_*re f l*_ in the Op paradigm showed a more systematic decrease as *L*_2_ increased, which was similar to the observation for the *I*/*O*_*gen*_. For the Sc paradigm, this reduction in the slope was only observed between low to mid levels and the slopes at high levels were very similar to mid levels due to the high saturation of *L*_*re f l*_. Accordingly, the Op paradigm better expressed the cochlear compressive behavior. In fact, the Op paradigm showed the plateau at high levels but the Sc paradigm plateaued at mid levels. Hence, the Op paradigm could provide more details about the *I*/*O*_*re f l*_ growth behavior and the cochlear compression. For future work, it will be important to compare these paradigms in participants with hearing loss (with different hearing thresholds).

## 5. Conclusions

This study found differences in the levels and I/O functions of the generator and reflection components obtained using three of the widely-used level-relation paradigms. The paradigms affected the overlap region of the nonlinear distortion component and accordingly affected the generator component. The impact of the different level-relation paradigms on the reflection components level was less noticeable as this component depends mainly on cochlear biomechanical characteristics at the reflection site. However, due to the impact of paradigms on the reflection component microstructure, the I/O functions were very much affected. The use of Op paradigm was found to be more beneficial for providing a more systematic generator/reflection component patterns and I/O functions with less variability across frequency.

## Figures and Tables

**Table 1.** The primary levels and frequency ranges of the three paradigms used in this study (i.e., Op, Sc, and EL). In all paradigms, *f*_2_/*f*_1_ = 1.22 and *L*_2_ changed in 5 *dB* increments. In the Sc paradigm, *L*_1_ = *L*_2_ for *L*_2_ ≥ 65 *dB SPL*.

Paradigm	Level Relation	*L*_2_	*f*_2_
Op	*L*_1_ = 0.45*L*_2_ + 44 *dB SPL*	25 – 75 *dB SPL*	*f*_2_ = 750 – 6000 *Hz*
Sc	*L*_1_ = 0.4*L*_2_ + 39 *dB SPL*	25 – 75 *dB SPL*	*f*_2_ = 1000 – 8000 *Hz*
EL	*L*_1_ = *L*_2_	40 – 75 *dB SPL*	*f*_2_ = 1000 – 8000 *Hz*

## Data Availability

Data from this study can be made available upon request to the corresponding author after executing appropriate data sharing agreement.

## Abbreviations

The following abbreviations are used in this manuscript:

ANOVA: Analysis of Variance
BM: Basilar Membrane
DPOAE: Distortion Product Otoacoustic Emission
EL: Equal-Level
I/O: Input/Output
Op: Optimal
Sc: Scissors
SNR: Signal to Noise Ratio
SOAE: Spontaneous Otoacoustic Emission
